# Data standards and interoperability for health

**DOI:** 10.1186/s12919-026-00396-y

**Published:** 2026-09-21

**Authors:** Barbara Tornimbene, Zoila Beatriz Leiva Rioja, Fenella Beynon, Jose Costa Teixeira, Agnes Kiragga, Gerard Krause, Michael Ochola, Sylvia Thun, Philippe Veltsos, Oliver Morgan

**Affiliations:** 1 World Health Organization Hub for Pandemic and Epidemic Intelligence, Berlin, Germany; 2 CPC Analytics, Berlin, Germany; 3 Swiss Tropical and Public Health Institute, Basel, Switzerland; 4 Digital Square, PATH, Washington, DC USA; 5 African Population and Health Research Center, Nairobi, Kenya; 6 World Health Organization Head Quarter, Geneva, Switzerland; 7 Berlin Institute of Health of the University Hospital Charité, Berlin, Germany

**Keywords:** Data standards, Interoperability, Pandemic intelligence, Public health surveillance, Digital health infrastructure

## Abstract

The need for improved pandemic and epidemic intelligence has underlined the significance of data standardisation in global health. This report examines the role of standardized frameworks in enabling seamless data exchange, facilitating real-time information sharing, and improving global coordination during public health emergencies. The COVID-19 pandemic underscored longstanding deficiencies in data interoperability that had already been identified in health systems and prior public health emergencies, both within and across countries. However, the pandemic brought unprecedented visibility and urgency to these issues, highlighting the critical need for global data standards such as HL7® FHIR® (Fast Healthcare Interoperability Resources), SNOMED CT, LOINC, and ICD. The adoption of these standards can enhance health systems' ability to exchange data promptly and accurately on an international scale, thereby improving preparedness and responsiveness.

Established guidance like the FAIR (Findable, Accessible, Interoperable, and Reusable) data principles and existing specifications like the International Patient Summary (IPS), among others, have supported the development of interoperable global health systems, lowered research redundancy and enhanced data quality. The adoption of such guidance and standards can contribute to enhancing scalable digital health initiatives and promoting international cooperation.

The report emphasizes the need for robust digital health infrastructure to address future pandemics. It underscores the continuous endeavours of organisations such as WHO (with the SMART guidelines initiative) and HL7 (with the standards like FHIR®) to standardise data sharing and enhance global health outcomes. Despite ongoing issues related to privacy and scalability, the implementation of global standards is essential for enhancing pandemic and epidemic intelligence and fortifying global health systems.

## Background

The rising demand for efficient pandemic and epidemic intelligence has highlighted the essential requirement for seamless data interchange throughout health systems and regions [1]. The COVID-19 pandemic brought greater attention to substantial deficiencies in early detection, response coordination, and resource allocation, mostly due to the lack of implementation of standardised data and interoperable systems [2, 3]. To tackle these difficulties, the development and adoption of global data standards is crucial, facilitating the rapid and precise exchange of health information among stakeholders [4, 5]. The use of established frameworks, particularly those designed for clinical data, offers a foundation for constructing a more cohesive and adaptive global health monitoring system. However, achieving this goal requires both the full implementation of existing standards and ongoing efforts to strengthen and expand these frameworks to better address future emergencies [6].

In this sense, standardization is crucial in healthcare and surveillance as it enhances communication and data exchange across various settings, ensuring smooth system integration and fostering effective global collaboration [7]. Standards facilitate interoperability, allowing diverse healthcare applications to work together seamlessly, a foundational element for global health initiatives. Beyond enabling efficient collaboration, standardized data exchange supports accurate assessment of population health needs, ensures quality data for individualized care across the care continuum, and enables the evaluation and improvement of healthcare services. Furthermore, technical platforms adhering to established standards and protocols promote robust data sharing and stakeholder engagement, overcoming political and logistical barriers [8–10].

In this session of the World Health Organization (WHO) Pandemic and Epidemic Intelligence Forum [11], co-hosted with the WHO Department of Surveillance Systems, the focus was on examining specific use of data standards and principles in public health, one component of promoting more effective information sharing and the impact of digital tools and international guidelines in ensuring effective information sharing. The objective was to explore existing solutions that can facilitate and support pandemic and epidemic intelligence. We brought the experience of the Berlin Institute of Health of the University Hospital Charité, Digital Square/the PATH Program, the Africa Population and Health Research Center, Digital Health Unit at the Swiss Tropical and Public Health (TPH) Institute to discuss the role of standardization in public health.

### The importance of principles for data standards and the role of global collaboration

Standardization enhances interoperability, efficiency, data quality, and the ability to address global health challenges, potentially leading to better health outcomes and more effective public health responses [5, 12]. It enables data exchange across diverse healthcare systems, ensuring data retains its correct meaning (semantics), consistency in expression (syntax), and allows for efficient retrieval (access). Aligning standardization processes with the FAIR (Findable, Accessible, Interoperable, Reusable) data principles is crucial to ensure scientific data can be efficiently used and shared [13, 14]. This can reduce the research waste – e.g. duplication of clinical trials—reduce costs and enhance the capacity to respond to global health crises by making data more accessible and usable, ultimately improving health outcomes.

To guide organizations in the adoption of a broad range of standards, several Standards Development Organizations created the Joint Initiative Council for Global Health Informatics Standardization (JIC), which is collaborating with the WHO since 2017 to enhance global health data standards [15]. JIC is a member of The Global Digital Health Partnership (GDHP), which supports the implementation of worldwide digital health services [16]. A notable JIC project is the International Patient Summary (IPS), which defines a core dataset and format for patient summaries, supporting continuity of care and unplanned cross-border healthcare scenarios [17]. IPS is being increasingly adopted by low- and middle-income countries, with several organizations like Africa CDC recommending its use where appropriate. While IPS provides a valuable international standard for sharing core patient information, it is not intended to replace the detailed data captured in full electronic health records (EHRs). Instead, IPS serves as a complementary tool, supporting continuity of care across borders and settings where access to complete EHR data may be limited. Additionally, the Global Alliance for Genomics and Health is developing standards for collecting, storing, analysing, and sharing genomic data, enabling an "internet of genomics” [18]. These actions are crucial for ensuring consistent, high-quality data exchange and interoperability across diverse healthcare systems, ultimately enhancing global health outcomes.

### What is already in use?

A key aspect of any digital health strategy is the effective selection and use of existing standards for different parts of the system, rather than the creation of custom solutions. Semantic standards like Systematized Nomenclature of Medicine—Clinical Terms (SNOMED), and the Logical Observation Identifiers Names and Codes (LOINC) help provide coherence in data semantics, with SNOMED CT offering a validated way to represent clinical information and LOINC facilitating the exchange of clinical results [19, 20]. The WHO’s International Classification of Diseases (ICD) ensures semantic consistency. These standards are integrated and built on other standards. For example, data exchange syntax is supported by standards like FHIR, which in turn use other standards like JavaScript Object Notation (JSON) and eXtended Markup Language (XML). On top of FHIR, which accommodates standards like SNOMED, LOINC and ICD, there are standard specifications like the IPS, and an increasing portfolio of guidance like that provided by Integrating the Healthcare Enterprise (IHE) [17, 21–24]. These widely used standards can thus be modularly leveraged and adapted to meet specific needs such as disease surveillance or clinical data exchange. This modular approach facilitates consistency and therefore supports interoperability, data quality, and ultimately the ability to address global health challenges, leading to better health outcomes and more effective public health responses.

In Europe, FHIR is promoted to support the European Health Data Space (EHDS), enabling data exchange while ensuring regulatory compliance [25, 26].

A national level example, Kenya’s Ministry of Health has integrated FHIR into its digital health strategy to ensure interoperability across various systems, demonstrating a high-level commitment to standards and enterprise architecture, which is essential for effective and long-lasting data exchange and health system integration. Institutions like the Berlin Institute of Health of the University Hospital Charité apply international standards to their digital health and interoperability projects. Charité’s use of FHIR, SNOMED CT, LOINC, and ICD-11 ensures structured and interoperable health data across different systems within Germany. By focusing on digital medicine and interoperability, these institutions play a crucial role in standardizing health data, developing frameworks for data integration, and facilitating international collaboration for improved health outcomes.

### Operationalization of standards: a challenging endeavour

Standards offer promising solutions but must be adaptable to different contexts, as healthcare processes and data requirements vary widely between and within countries and systems. Low and middle-income countries often face challenges in adopting these standards due to resource constraints. Implementing these standards involves overcoming costs and learning curves, and while developing bespoke solutions may be easier for short-term goals, it undermines long-term interoperability and data consistency.

In response to this challenge, the PATH Program initiative, Digital Square, supports the development of a global digital public infrastructure by matching the supply and demand for digital public goods and health solutions. PATH aims to improve health workers' access to information and enable countries to strengthen their healthcare capacities and infrastructures. It coordinates efforts among investors and government bodies to ensure a structured and effective approach to healthcare improvement, promotes open-source solutions through the Global Goods Guidebook and other initiatives, and works with regional bodies to enhance digital transformation, applying the Principles for Digital Development [27]. These principles support making designs so that systems are scalable, collaborative, avoiding digitalization to consist of isolated pilot projects.

A prime example of the operationalization of a standardized approach to implementing health interventions globally are the WHO's SMART guidelines [28]. Both PATH and The Digital Health Unit at Swiss TPH, a global health institute dedicated to improving health and wellbeing through excellence in research, have collaborated with WHO on their development. The WHO's SMART guidelines provide a comprehensive framework for standardizing healthcare data and processes, covering both semantic and syntactic aspects. They define essential data elements and standardized activities like patient registration, ensuring uniform expression across systems for consistency and interoperability. A key strength of these standards lies in the extensive and rigorous process of transforming clinical and operational guidance into digital components, a complex, lengthy, and often error-prone task requiring substantial investment and specialized skills. Although WHO has supported national adaptations in some contexts, fully detailed materials and tools to facilitate adaptation and effective implementation across various settings are still limited, with further resources expected to be developed.

### Integration vs interoperability

Effective standardization requires distinguishing between integration and interoperability. Integration creates direct, point-to-point connections between systems, which are complex and costly to maintain, as each update necessitates adjustments to these connections. In contrast, interoperability uses standards and middleware to facilitate data flow without direct connections, reducing complexity and costs, and ensuring efficient data sharing at national, regional, and global levels.

Achieving interoperability is challenging, particularly in countries with disparate systems. It involves understanding various steps and actors, covering technical, syntactic, semantic, and organizational aspects. Currently, interoperability is organized according to the International Organization for Standardization Open Systems Interconnection (ISO OSI) model [29]. This framework divides network communication into seven layers, each with specific functions. The application-level layer seven (HL7) plays a crucial role in enabling the seamless integration of healthcare systems worldwide [30].

In this context, HL7 International's collaboration with the Observational Health Data Sciences and Informatics (OHDSI) aims to provide a single common data model for sharing information in clinical care and observational research [31]. Notably, the OMOP Common Data Model has been recognized as a digital public good through efforts in collaboration with Digital Square and the OHDSI Africa Chapter. OHDSI is a multi-stakeholder, interdisciplinary collaboration focused on unlocking health data value through large-scale analytics and open-source solutions. OHDSI vocabularies standardize medical terms across various clinical domains for the Observational Medical Outcomes Partnership (OMOP) Common Data Model (CDM) [32]. OMOP, supported by an international network of collaborators, enables the integration and analysis of health data from diverse settings, contributing to a comprehensive understanding of health outcomes and disease patterns worldwide.

Aside from following those available frameworks, establishing interoperability begins with a comprehensive digital health strategy that includes the right enterprise architecture. According to PATH, this strategy should define standards with specific data and purposes in mind before implementation, avoiding the need for multiple disease-specific structures. Following the WHO's Digital Implementation Investment Guide, which recommends defining standards and interoperability from the outset, can help streamline the process.

### Creating networks that support standardization

From a regional context, the APHRC, based in Kenya and with a regional office in Senegal, aims to harmonize healthcare data across Africa. APHRC's mission is to transform lives through research that informs policy and actionable activities to improve health and development on the continent. Their data science program focuses on building platforms and architectures to support data ecosystems in Africa. This includes creating systems for data sharing, harmonizing datasets, and building capacity for data management, while exploring big data analytics, AI, machine learning, and generative AI in healthcare.

One of APHRC's key initiatives is the Implementation Network for Sharing Population Health Information from Research Entities (INSPIRE Network), which uses demographic and health surveillance sites across Africa to collect and analyse health data [14, 33]. This network aims to make data FAIR” by using standards to harmonize data from different communities, contributing to sustainable development goals and global health literacy [14]. INSPIRE integrates various datasets, including Ministry of Health data, disease registries, and research projects, into a common data model using standards like SNOMED CT, LOINC, and ICD-11, facilitating efficient data flow through standards and middleware.

In Germany, the Medical Informatics Initiative is a significant national project involving all German university hospitals collaborating with research institutions, industry, health insurers, and patient advocacy groups. This initiative aims to leverage research findings for direct patient benefit. Challenges include managing routine clinical data, patient consent, data access governance, structured clinical data sets, and implementing and analysing these data sets. To address these, they have created a nationwide infrastructure of data integration centres following existing standards and a core dataset based on the IPS specification. Their interoperability working group coordinates platform agreements and concrete steps for achieving interoperability, such as harmonizing data structures and conducting federated analysis on patient data.

### Example for harmonize data sharing in Germany

With a similar goal, the University Hospital Charité developed the German Corona Consensus Dataset (GECCO) during the pandemic, a standardized dataset for COVID-19 research defined by a multidisciplinary expert board that uses international standards and terminologies [34]. This was followed by the microbiology module of the Medical Informatics Initiative. Additionally, they created portals such as the German Portal for Medical Research Data, the Medical Informatics Initiative Structured Pathology Findings, and the Medical Informatics Initiative Molecular Genetic Findings. These platforms allow German scientists to query data availability and request usage, with approvals reviewed by access committees at each participating university hospital.

Germany also developed the “phenopackets” specification, a schema that supports the exchange of computable longitudinal case-level phenotypic information for diagnosing and researching various diseases. This includes complex genetic diseases, cancer, infectious diseases, signs, symptoms, laboratory and imaging findings, and physiological tests. Furthermore, the NFDI4HEALTH initiative was launched to create a comprehensive inventory of German epidemiological, public health, and clinical trial data, establishing a centralized data catalogue with advanced search functionalities, sophisticated data access management, and a data analysis toolbox, while adhering to stringent privacy requirements [35].

Similar to the GECCO experience, APHRC engaged in the PEACH Project (Platform for Evaluation and Analysis of COVID-19 Harmonized Data) during the pandemic, aiming to create an integrated platform for decision-making and research using harmonized COVID-19 data from Kenya, Uganda, and Malawi [36]. This project focused on creating common data standards and utilizing tools like OMOP CDM to harmonize and analyse health data. Despite the challenges of diverse data protection frameworks and the need for capacity building, APHRC advocated for federated platforms and international standards, adapting them to the African context. Effective data governance and trust-building among data collectors, users, and governors are essential for successful data harmonization and improved health outcomes in Africa.

Both Charité and APHRC leverage federated platforms for data harmonization and analysis, enhancing data security and privacy while allowing for the generation of comprehensive results.

### Standardization in clinical settings

Achieving interoperability and standards in clinical settings is crucial for securing the relationship between clinical interactions and surveillance. Quality surveillance data often originates from clinical interactions, making it essential to ensure that these interactions capture complete and accurate information. By adopting a 'collect once, use many' approach, this information can be efficiently utilized across multiple systems and contexts, ensuring it is quickly and accurately transferred to surveillance systems to support public health decision-making. However, this process faces numerous challenges, especially in resource-constrained settings. Often, only minimal data from clinical interactions is recorded (e.g. only ‘diagnosis’), whether on paper or digitally. Health workers’ abilities to capture accurate clinical information may be constrained by limited clinical skills or access to diagnostics, challenges in guideline adherence due to a wide range of personal, guideline-related and external factors, or issues in transcription, particularly to digital systems in the context of low digital literacy [37]. These factors vary substantially within and between clinical settings, creating challenges for comparability – for example between primary and secondary or tertiary facilities, or with differences in diagnostic availability over time. Studies of data quality in clinical settings highlight data discrepancies between clinical encounters and recorded data, with multifaceted challenges relevant to surveillance, including those of accuracy, consistency, completeness, contextual validity, accessibility and currency [38, 39]. Therefore, addressing both the challenges of quality clinical encounters, and the entry of data into digital systems for clinical care which can be reused for surveillance, is critical.

The focuses is on improving the quality of care through digital tools that provide real-time decision support to healthcare providers. Clinical Decision Support (CDS) and Clinical Decision Support Systems (CDSS) are digital tools that help healthcare providers adhere to evidence-based recommendations, by providing them with patient-tailored recommendations based on clinical algorithms combined with individual data. CDSS can range from simple alerts to comprehensive consultation support and can improve guideline adherence and data quality. For example, while paper-based clinical records often lack detail, digital systems can capture comprehensive data during consultations. Whilst other digital systems, such as electronic medical records, can carry out this function, CDSS can offer additional benefits in terms of accuracy of clinical assessment, diagnosis and management, and completeness, by incentivizing use through functions such as automating calculations required for clinical care. Provided CDSS data is captured according to standards, it can allow for timely re-use within surveillance systems and enable better comparability (for example, rather than only being able to compare ‘pneumonia’, to be able to compare each component of the case definition, which may differ between settings and over time).

### Good governance and capacity building for standardised digital health

PATH Digital Square initiative aims to make adopting standards more feasible, even in resource-constrained settings, by engaging local stakeholders, offering training and support, and demonstrating the long-term benefits of established standards for better health outcomes. For instance, the WHO's Digital Implementation Investment Guide helps define standards and interoperability from the outset. This involves starting with a clear digital health strategy, establishing the right enterprise architecture, and ensuring data standards are purpose-designed before implementing disease-specific projects.

Standardized roles are necessary to support the adoption of standards, as expertise varies significantly across regions. Key roles include implementers, clinical authors, technical authors, and architects, each essential for different levels of standardization and technical specifications. Project managers also ensure timelines are met and projects stay on track. The APHRC focuses on building local capacity for data management and analysis by training researchers and health professionals in data harmonization, governance, and the use of advanced analytical tools like machine learning and AI. The center promotes local data repositories and establishes data governance frameworks to ensure data privacy and security while encouraging data sharing for research and public health purposes. More importantly, the team has underscored the need to customize different data standards to incorporate Africa-specific data concept in international vocabularies. The diversity of African settings, including over 2000 languages in the continent, calls for continuous modifications in key concepts to support data harmonization in Africa.

Developing a comprehensive capacity-building framework equips individuals and organizations with the skills and knowledge necessary to adopt standards, enhancing disease surveillance and data integration efforts. This approach emphasizes collaboration among various stakeholders to improve data accessibility and quality, aligning global efforts and fostering a culture of data sharing and cooperation.

## Future perspectives and open questions

The work of organizations like Charité University, the PATH Program, APHRC, Swiss TPH and WHO shows the future of global health projects. Several future viewpoints surface as these initiatives keep developing and using digital health solutions.

One important viewpoint is the acceptance and progress toward interoperability criteria. Enhanced quality and timeliness of data can result from better interoperability between several health information systems. Great promise exists in incorporating artificial intelligence and machine learning into clinical decision support systems to enhance public health surveillance. By leveraging large datasets and real-time inputs, these technologies can support earlier detection of outbreaks, identify warning signs, and prompt timely interventions to mitigate public health risks. Another important emphasis is on broadening the use of advanced digital health instruments via means of capacity development. Designed to handle many facets of healthcare from diagnosis to treatment and follow-up, these technologies will probably get more user-friendly, accessible, and scalable. Especially crucial for improving healthcare delivery in environments with limited resources are such developments.

The relevance of data standards for the advancement of pandemic and epidemic intelligence is a critical lesson to be learnt from this forum. The adoption and adaptation of global standards can considerably enhance the quality, consistency, and accessibility of health data, as evidenced by the lessons learnt from clinical data standardisation, such as those found in SNOMED CT, LOINC, and ICD. ICD-11, with its enhanced digital compatibility, expanded classification system, and updated medical terminology, significantly differs from ICD-10, addressing many limitations of its predecessor. These improvements, including greater granularity and adaptability for local contexts, are well-positioned to help overcome challenges discussed in this report, such as data standardization, interoperability, and the integration of health information systems across diverse setting. Real-time data sharing and decision-making across borders can be facilitated by these standards, which can serve as a robust foundation for pandemic intelligence. For example, the integration of clinical and surveillance data through the use of standardised frameworks enables the more efficient identification of emergent threats, a more coordinated response, and improved monitoring of health outcomes across various regions.

In order to establish a resilient pandemic intelligence infrastructure, it is imperative to develop interoperable systems and implement global data standards. Global health stakeholders can guarantee that critical information is seamlessly transferred between clinical, research, and public health systems by utilising existing frameworks, such as the FAIR principles and initiatives like the International Patient Summary (IPS). This will facilitate more rapid and precise responses to health emergencies, particularly in low- and middle-income countries where the digital health infrastructure is still in the process of developing. In order to fully realise the potential of data standards in pandemic and epidemic intelligence, it will be imperative to address challenges such as scalability, privacy, and security while coordinating efforts across various regions. In the end, these endeavours will contribute to the development of a more resilient global health system that is capable of predicting and mitigating future health crises.

## Abbreviations

WHO: World Health Organization
HL7: Health Level Seven International
FHIR: Fast Healthcare Interoperability Resources
SNOMED CT: Systematized Nomenclature of Medicine—Clinical Terms
LOINC: Logical Observation Identifiers Names and Codes
ICD: International Classification of Diseases
FAIR: Findable, Accessible, Interoperable, and Reusable
IPS: International Patient Summary
OMOP: Observational Medical Outcomes Partnership
OHDSI: Observational Health Data Sciences and Informatics
